# The Incidence of Serious/Invasive Bacterial Diseases in Infants 90 Days Old or Younger at an Emergency Hospital in Japan

**DOI:** 10.7759/cureus.36494

**Published:** 2023-03-21

**Authors:** Saeka Yoshitake, Yoshiki Kusama, Kenta Ito, Hiroyuki Kuroda, Muneyasu Yamaji, Kento Ishitani, Yusuke Ito, Katsunori Kamimura, Toshiro Maihara

**Affiliations:** 1 Department of Pediatrics, Hyogo Prefectural Amagasaki Medical Center, Amagasaki, JPN; 2 Department of Pediatrics, Hyogo Prefectural Amagasaki General Medical Center, Amagasaki, JPN; 3 Department of General Pediatrics, Aichi Children’s Health and Medicine Hospital, Obu, JPN; 4 Department of Pediatric Infectious Diseases, Hyogo Prefectural Amagasaki Medical Center, Amagasaki, JPN; 5 Department pf Pediatrics, Hyogo Prefectural Amagasaki General Medical Center, Amagasaki, JPN

**Keywords:** incidience, infantile fever, invasive bacterial infection, severe bacterial infection, lumbar puncture

## Abstract

Background

The incidence of severe bacterial infections (SBIs) in infants aged ≤90 days is thought to have decreased because of widespread vaccination programs. However, relevant epidemiological data in Japan are scarce.

Materials and methods

This observational, single-center study investigated the epidemiology of fever in infants aged ≤90 days. SBI was defined as the presence of meningitis, urinary tract infections (UTIs), or bacteremia. Invasive bacterial infection (IBI) was defined as the presence of meningitis, bacteremic UTI, or bacteremia. We determined the incidence of UTIs, bacteremia, meningitis, SBIs, and IBIs in the following three age groups: 0-28, 29-60, and 61-90 days. We subsequently calculated the relative incidence for the groups aged 29-60 and 61-90 days, using the group aged 0-28 days as the reference group.

Results

Herein, 58, 124, and 166 infants were included in the 0-28 days, 29-60 days, and 61-90 days age groups, respectively. Of the total number of patients, 15.5%, 8.9%, and 16.9% in the 0-28 days, 29-60 days, and 61-90 days age groups, respectively, were diagnosed with SBI. The relative incidences were 1 for the 0-28 days group (reference group), 0.67 for the 29-60 days group (95% confidence interval [CI], 0.39-1.15), and 1.08 for the 61-90 days group (95% CI, 0.58-2.00). Of the total number of patients, 10.3%, 3.2%, and 0.6% in the 0-28 days, 29-60 days, and 61-90 days age groups, respectively, were diagnosed with IBI. Relative incidences were 1 (reference group), 0.50 (95% CI, 0.29-0.88), and 0.28 (95% CI, 0.19-0.41) for the 0-28 days, 29-60 days, and 61-90 days age groups, respectively. All cases of IBI were caused by Group B streptococcus (GBS), except for two cases of bacteremia, which were caused by *Haemophilus influenzae*.

Conclusion

The incidence of SBI was similar in the 0-28 days and 61-90 days age groups. However, the incidence of IBI decreased with increasing age. The incidence of UTIs was highest in the 61-90 days age group, and that of meningitis and bacteremia decreased with increasing age.

## Introduction

Before the widespread use of pneumococcal vaccines, infants aged ≤90 days with fever were considered to be at high risk for serious bacterial infection (SBI). These patients routinely underwent a full sepsis workup, including urine, blood, and cerebrospinal fluid (CSF) culture [1], a practice that is recommended to date by some pediatricians. The epidemiology of SBI has changed dramatically in response to widespread pneumococcal and *Haemophilus influenzae* type B (Hib) vaccination programs [2,3]. Three studies evaluating the epidemiology of infantile fever demonstrated the decline of bacteremia in these patients. SBIs are now less commonly observed in infants older than one month except for urinary tract infections (UTIs) [4-6]. It has also been reported that the incidence of bacteremia caused by pneumococcal or Hib infection has decreased in Japan in the past decade due to widespread immunization programs [7,8]. Therefore, the epidemiology of fever in infants aged ≤90 days in Japan is considered to be similar to that reported in the United States. However, to our knowledge, no epidemiological studies have focused on the etiology of fever in this age group in Japan. Herein, we investigated the incidence of SBI in infants aged ≤90 days who presented at our hospital with a fever.

## Materials and methods

Design

This single-center observational study involved the analysis of data obtained from electronic medical records.

Setting

This study was performed at the Hyogo Prefectural Amagasaki General Medical Center, a pediatric emergency and critical care center that provides primary and tertiary care in the Hanshin area. It provides medical care for children who reside in Amagasaki city (child population: 60,000), Nishinomiya city (child population: 70,000), and Takarazuka city (child population: 32,000). In 2020, the hospital provided medical care to 23,277 pediatric patients, 921 as inpatients and 22,356 as outpatients.

Participants

Febrile infants aged ≤90 days who visited our outpatient clinics or emergency department between January 1, 2016, and December 31, 2020, were enrolled in the study. We divided the infants into three groups based on age: 0-28, 29-60, and 61-90 days. These categories were devised according to the clinical practice guidelines developed by the American Academy of Pediatrics [6]. Data from January 2021 to December 2022 were excluded to eliminate the effects of the coronavirus disease 2019 outbreak (The first pediatric case of COVID-19 presented to our hospital in 2021.). Fever was defined as an axillary temperature ≥38.0°C. The exclusion criteria were as follows: infants who visited our hospital for a routine health check or immunization and presented with accidental hyperthermia; patients who were transferred to another hospital on the day of the visit; and patients with serious underlying diseases, such as severe neurological or cardiovascular abnormalities or immunodeficiency.

Definitions

Meningitis was diagnosed by a positive CSF culture and/or increased cell count in CSF and a positive blood culture that isolated pathogens suspected of causing meningitis. UTIs were diagnosed by a positive urinary leukocyte esterase reaction with a positive urine culture. A urine culture was deemed positive if a single bacterial species was detected at >10^4^ CFU/mL in the urine, which was captured using a sterile urinary catheter. Bacteremia was diagnosed by a positive blood culture that was judged not to be caused by contamination on clinical grounds. Meningitis with bacteremia and UTI with bacteremia were not classified as bacteremia. UTIs were classified as bacteremic UTIs and non-bacteremic UTIs. SBI was defined as the presence of meningitis, UTI, or bacteremia, and invasive bacterial infection (IBI) was defined as the presence of meningitis, bacteremic UTI, or bacteremia. Classifications of SBI and IBI were based on the previous reports [9-11].

Extracted data

The following information was extracted from the electronic records: date of birth; age (days); sex; hospitalization; results of blood, urine, and spinal fluid cultures; and bacteria isolated from the cultures.

Outcome measures

We determined the incidence of UTIs, bacteremia, meningitis, SBIs, and IBIs in the following three age groups: 0-28, 29-60, and 61-90 days. We subsequently calculated the relative incidence for the groups aged 29-60 and 61-90 days, using the group aged 0-28 days as the reference group.

Statistical analysis

The results were statistically assessed using Fisher's exact test. Significance was defined as a p-value <0.05.

Ethics statement

This study was approved by the ethics committee of the Hyogo Prefectural Amagasaki General Medical Center. Consent was obtained via the opt-out method due to the retrospective nature of the study.

## Results

The demographic and clinical characteristics of each age group are presented in Table 1. Blood cultures were performed for more than 80% of infants in each age group. However, the proportion of infants who underwent urine and CSF cultures decreased with increasing age (81.0%, 75.0%, and 68.1% for urine cultures and 60.3%, 39.5%, and 27.7% for CSF cultures, in the 0-28 days, 29-60 days, and 61-90 days age groups, respectively). Of the total number of patients, 15.5%, 8.9%, and 16.9% in the 0-28 days, 29-60 days, and 61-90 days age groups, respectively, were diagnosed with SBI. The relative incidences were 1 for the 0-28 days group (reference group), 0.67 for the 29-60 days group (95% confidence interval [CI], 0.39-1.15), and 1.08 for the 61-90 days group (95% CI, 0.58-2.00). The lowest frequency of SBIs was observed in the 29-60 days age group, though the difference in incidence was not statistically significant. In contrast, the proportions of IBI were 10.3%, 3.2%, and 0.6%, and the relative incidences were 1 (reference group), 0.50 (95% CI, 0.29-0.88), and 0.28 (95% CI, 0.19-0.41) for the 0-28 days, 29-60 days, and 61-90 days age groups, respectively. The proportion of IBI decreased with increasing age. The incidence of IBI in the 61-90 days group was significantly lower than that in the 0-28 days group.

**Table 1:** Patient demographic and clinical characteristics CI, confidence interval; IBI, invasive bacterial infection; RI, relative incidence; SBI, serious bacterial infection; UTIs, urinary tract infections. SBI: meningitis plus bacteremia plus UTIs, irrespective of co-existing bacteremia. IBI: meningitis plus bacteremia plus bacteremic UTIs. Blood culture, urine culture, and lumbar puncture represent the number of patients for whom each test was obtained. Asterisks represent p <0.05 for Fisher's exact test.

Age group (days)	0-28 (n = 58)	29-60 (n = 124)	61-90 (n = 166)
Male (%)	30 (51.7)	68 (54.8)	99 (59.6)
Female (%)	28 (48.3)	56 (45.2)	67 (40.4)
Age (days (quartile))	17.5 (11, 23)	45 (38, 54)	75 (67, 81)
Hospitalization (%)	53 (91.4)	111 (89.5)	135 (81.3)
Blood culture (%)	49 (84.5)	108 (87.1)	144 (86.7)
Urine culture (%)	47 (81.0)	93 (75.0)	113 (68.1)
Lumbar puncture (%)	35 (60.3)	49 (39.5)	46 (27.7)
UTIs	3 (5.2)	8 (6.5)	27 (16.3)
RI (95% CI)	1 (reference)	1.18 (0.44-3.17)	2.84 (0.95-8.49)
Bacteremic UTIs (%)	0 (0)	1 (0.8)	0 (0)
Meningitis	3 (5.2)	2 (1.6)	0 (0.0)
RI (95% CI)	1 (reference)	0.52 (0.24, 1.09)	0.25 (0.20, 0.31)*
Bacteremia	3 (5.2)	1 (0.8)	1 (0.6)
RI (95% CI)	1 (reference)	0.41 (0.22, 0.76)	0.33 (0.18, 0.61)
SBI	9 (15.5)	11 (8.9)	28 (16.9)
RI (95% CI)	1 (reference)	0.67 (0.39, 1.15)	1.08 (0.58, 2.00)
IBI	6 (10.3)	4 (3.2)	1 (0.6)
RI (95% CI)	1 (reference)	0.50 (0.29, 0.88)	0.28 (0.19, 0.41)*

**Figure 1:**
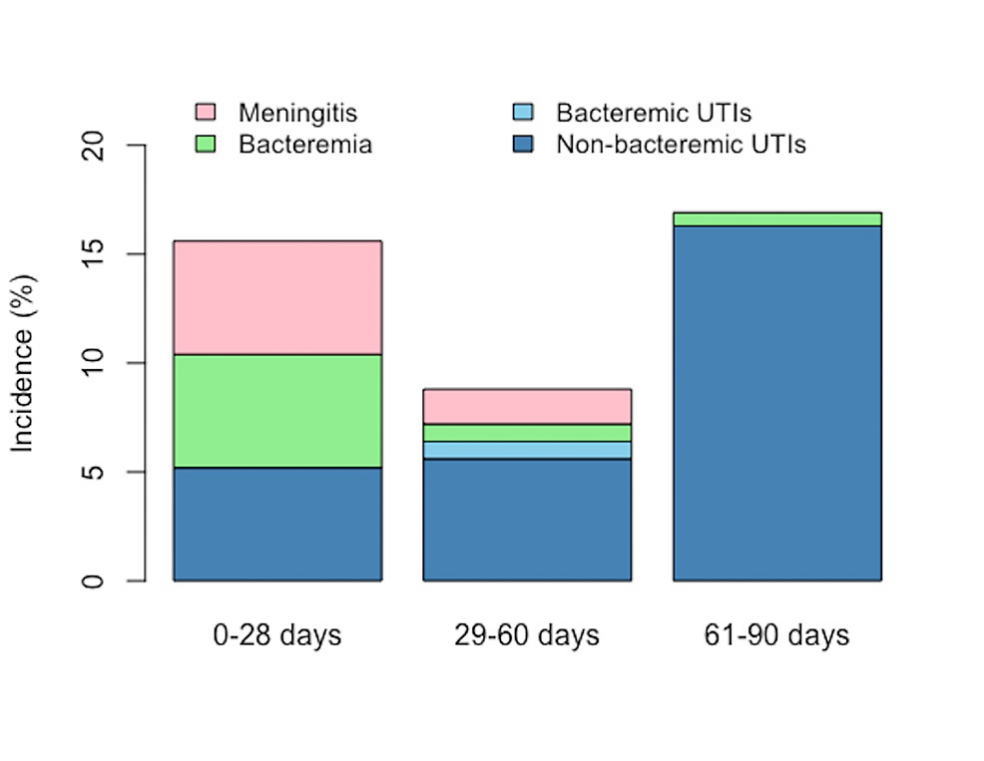
Incidence of meningitis, bacteremia, and UTIs according to age groups UTI, urinary tract infection.

Further stratifications of the incidence of SBI according to diseases are illustrated in Figure 1. The incidences of meningitis and bacteremia were both 5.2% in the 0-28 days age group; the incidences decreased with increasing age. In the 61-90 days age group, only one patient presented with bacteremia, and no patients presented with meningitis or bacteremic UTIs. However, 27 patients presented with UTIs. The incidence of UTIs was higher in the 61-90 days age group compared with other age groups, although the difference was not significant.

Bacteria isolated from the cultures are presented in Table 2. Among the 10 cases with IBI, the causative bacteria were identified as Group B streptococcus (GBS) in 8 (80%) cases. GBS was also identified as the causative agent in all cases diagnosed with meningitis. No pneumococci were isolated from cultures obtained in this study. In two cases, *H. influenzae* were isolated in the cultures obtained from patients with bacteremia.

**Table 2:** Isolated bacteria from urine, blood, and cerebrospinal fluid according to age groups GBS: Group B streptococcus; UTI, urinary tract infection. One UTI in the 29-60 days group with an asterisk accompanied bacteremia.

Age group	0-28 days	29-60 days	61-90 days
Meningitis	GBS 3	GBS 2	
Bacteremia	GBS 3	*Haemophilus influenzae* 1	*Haemophilus influenzae* 1
UTIs	*Escherichia coli* 2, *Enterococcus faecalis* 1	*Escherichia coli* 7, *Enterococcus faecalis* 1*	*Escherichia coli *16*, Enterococcus faecalis* 4, *Klebsiella pneumoniae* 3, *Klebsiella oxytoca* 1, *Klebsiella aerogenes *1, GBS 1

## Discussion

The epidemiology of fever in infants aged ≤90 days has been mainly investigated in the US and in European countries, with only a few relevant studies conducted in Japan [12-14]. Nomura et al. investigated the risk factors for SBI in infants aged ≤90 days but did not report any age-specific epidemiology [12].

The American Academy of Pediatrics developed the guidelines for the evaluation and management of febrile infants in 2021 [15]. The guidelines referred to representative studies regarding the incidence of febrile infants [4-6], all of which had demonstrated an age-dependent decrease in the incidence of IBI. In our study, although the incidence of SBI was similar in the 0-28 days and 61-90 days age groups, the incidence of IBI decreased with increasing age. Hence, our study has demonstrated that the incidence of IBI in the Amagasaki region demonstrated similar trends as reported by other studies. 

The epidemiology of UTIs in infants ≤90 days of age is not well known. In our study, the incidence of UTIs was the highest in the 61-90 days age group. A previous single-center retrospective study investigating UTIs in infants ≤3 months old demonstrated a median age of 63 days [16] and consistent trends in epidemiology. In contrast to the incidence of UTIs, the incidences of meningitis and bacteremia decreased with increasing age. This trend can be explained by bacterial etiology. In our study, all cases of IBI were caused by GBS, except for two cases diagnosed with bacteremia. Although pneumococcus and Hib were the two most common causes of meningitis and bacteremia before the initiation of universal pneumococcal and Hib vaccination programs, GBS is the leading cause of these diseases currently [17]. Recent epidemiological data from Japan demonstrated that the median (interquartile range) patient age at diagnosis was 27 (17-45) days for late-onset GBS infections (i.e., occurring in patients aged <90 days) [18]. Therefore, IBI is considered to be rare in infants aged 61-90 days. Despite the implementation of a universal immunization program for Hib in Japan, the causal bacteria were identified to be *H. influenzae* in two cases of bacteremia in the 29-60 days and the 61-90 days age groups. However, it could not be ascertained if the bacteria were Hib due to a lack of serological testing.

In our study, there were no cases of meningitis in patients aged 61-90 days. A previous study has also demonstrated a low incidence of meningitis in patients aged 61-90 days [11]. The findings from our study were consistent with these results. Injudicious use of lumbar puncture to exclude meningitis is associated with complications such as lumbar puncture-associated infection and epitheliomas [19]. It is also associated with increased medical costs arising from unnecessary hospitalization [20]. Recent data have demonstrated that lumbar punctures in febrile infants aged >60 days are commonly performed in some hospitals in Japan [21]. However, further large-scale studies are required to clarify the indications to perform lumbar punctures for the diagnosis of meningitis.

This study has some limitations. First, this was a single-center study; therefore, it is unclear whether the findings can be generalized to other hospitals. Second, due to the retrospective nature of this study, information biases could have influenced the results. Third, we included our study population on the basis of the presence of fever, which was defined as an axillary temperature ≥38.0°C, at the time of the emergency room or outpatient clinic visit. Hence, we may have missed cases of infants with sepsis presenting with lower body temperatures. Fourth, it is possible that infants with SBI, who recovered naturally and did not have cultures performed, were excluded. Finally, only 53.1% of infants, who did not undergo hospitalization, were followed up at our hospital; hence, information regarding these infants may be incomplete. However, as our hospital is the largest pediatric emergency and critical care center in the area, it is expected that most infants would have returned to this hospital if their conditions had worsened.

## Conclusions

We investigated the epidemiology of fever in infants younger than three months of age and observed that the incidence of SBI was similar in the 0-28 days and 61-90 days age groups. However, the incidence of IBI decreased with increasing age. The incidence of UTIs was highest in the 61-90 days age group, and that of meningitis and bacteremia decreased with increasing age. All cases of IBI were caused by GBS, except for two cases of bacteremia, which were caused by *H. influenzae*. Our study has provided important information regarding the incidence of SBIs/IBIs in Japan.
